# Mental Fatigue in Collegiate Athletes: A Behavioral Science Review of Stress Appraisal, Competitive Anxiety, and Resilience-Related Regulation

**DOI:** 10.3390/bs16071133

**Published:** 2026-07-06

**Authors:** Zihan Gao, Wan Ahmad Munsif Wan Pa, Mohamad Nizam bin Nazarudin

**Affiliations:** Faculty of Education, Universiti Kebangsaan Malaysia, Bangi 43600, Malaysia

**Keywords:** mental fatigue, collegiate athletes, student-athletes, perceived stress, competitive anxiety, psychological resilience, behavioral science

## Abstract

Mental fatigue is an increasingly important concern among collegiate athletes whose academic–athletic roles require sustained cognitive effort, emotional regulation, and recovery across overlapping performance contexts. This structured narrative review synthesizes behavioral science, sport psychology, and athlete mental health literature to clarify how perceived stress, competitive anxiety, and psychological resilience may interact in the development and regulation of mental fatigue among collegiate athletes. Rather than treating mental fatigue as simple tiredness or an isolated performance symptom, this review conceptualizes it as a cognitive–emotional and psychobiological outcome shaped by stress appraisal, attentional load, effort regulation, and resource depletion. The main contribution of this review is to integrate previously separate lines of research into a collegiate-athlete-focused behavioral science framework in which perceived stress is positioned as an upstream appraisal-based condition, competitive anxiety as a proximal emotional mechanism, and psychological resilience as a dynamic regulatory resource that may buffer fatigue-related vulnerability. Tennis and Chinese/non-Western collegiate sport contexts are used as illustrative applications rather than exclusive empirical targets, highlighting how individual accountability, academic–athletic role demands, cultural expectations, and support structures may shape fatigue processes. This review also distinguishes established empirical evidence from theoretical inference and identifies key gaps in measurement heterogeneity, methodological transparency, longitudinal evidence, and culturally diverse collegiate athlete research. By refining the stress–anxiety–fatigue pathway and identifying resilience, recovery, and contextual support as important regulatory factors, this review provides a conceptual foundation for future empirical testing and for more targeted behavioral interventions to support collegiate athlete well-being and performance sustainability.

## 1. Introduction

Collegiate athletes occupy a demanding dual-role environment in which academic responsibilities and athletic expectations often coexist rather than occur separately. Unlike non-athlete students, they are required to maintain academic progress while also meeting training schedules, competitive demands, travel requirements, institutional expectations, and performance evaluation. These overlapping demands create a sustained psychological context in which attention, emotional regulation, coping capacity, and recovery must be repeatedly mobilized across educational and sport-related settings (Lu et al., 2012; Stambulova & Wylleman, 2019; van Rens et al., 2019). From a behavioral science perspective, this environment provides an important context for examining how stress appraisal, anxiety responses, self-regulation, and cognitive–emotional depletion may interact in shaping athlete well-being and performance sustainability.

Mental fatigue is particularly relevant within this context because it reflects more than a general sense of tiredness. It is commonly conceptualized as a psychobiological state that arises after prolonged cognitive effort and is associated with reduced motivation, impaired attentional control, slower decision-making, increased perceived effort, and weakened self-regulatory capacity (Marcora et al., 2009; Van Cutsem et al., 2017; Pageaux & Lepers, 2018). In collegiate athletes, mental fatigue may be shaped not only by sport-specific cognitive demands but also by academic workload, role conflict, emotional pressure, and repeated performance evaluation. This distinction is important because athletes may remain physically capable while still experiencing difficulty sustaining concentration, emotional stability, and decision quality under pressure. Existing sport-related reviews further suggest that mental fatigue can impair physical and skilled performance, especially when tasks require sustained attention, executive control, and rapid decision-making (Habay et al., 2021; Sun et al., 2021).

Although mental fatigue has clear relevance across collegiate sport, it should not be viewed as an isolated outcome. Rather, it is likely shaped by broader psychological processes that influence how athletes interpret, respond to, and recover from demands. Perceived stress is one such upstream process. From the perspective of the Transactional Model of Stress and Coping, stress emerges when individuals appraise environmental demands as threatening, excessive, or beyond their available coping resources (Lazarus & Folkman, 1984). In collegiate sport, perceived stress may arise from academic deadlines, training load, selection pressure, performance expectations, social evaluation, and uncertainty about future development. When these demands are repeatedly appraised as difficult to control, athletes may experience sustained vigilance, worry, and self-monitoring, which can gradually consume cognitive and emotional resources. This process is consistent with theoretical models that position perceived stress as an upstream appraisal-based condition contributing to competitive anxiety and mental fatigue.

Competitive anxiety may represent a more proximal mechanism through which broader stress appraisals become fatigue-related impairment. Competitive anxiety is generally understood as a psychological and psychophysiological state involving worry, apprehension, tension, and heightened arousal in evaluative performance situations (Woodman & Hardy, 2003). Unlike perceived stress, which reflects a broader appraisal of ongoing demands, competitive anxiety is more closely tied to immediate performance contexts. It may disrupt attentional control, increase intrusive thoughts, heighten physiological activation, and make effective decision-making more difficult. In cognitively demanding sports, these anxiety-related processes may increase the mental effort required to maintain performance, thereby contributing to mental fatigue over time. Research on perfectionism and competitive anxiety further suggests that athletes who react strongly to mistakes or perceived failure may experience greater psychological cost during competition (Stoeber et al., 2007; Rice et al., 2019).

Psychological resilience is important because athletes exposed to similar demands do not necessarily experience the same degree of fatigue-related impairment. Resilience is commonly understood as the capacity to adapt positively to adversity, recover from stress, and maintain functional stability under pressure (Fletcher & Sarkar, 2012, 2013). Rather than being treated only as a fixed personality trait, resilience is increasingly conceptualized as a dynamic and developable regulatory resource that shapes how individuals appraise stressors, select coping strategies, and recover from strain. This interpretation is consistent with Conservation of Resources theory, which argues that individuals strive to acquire, protect, and replenish valued resources, and that stress becomes harmful when those resources are threatened or depleted (Hobfoll, 1989). Within this framework, resilience may reduce the likelihood that perceived stress and competitive anxiety are translated into sustained mental fatigue by supporting adaptive appraisal, emotional recovery, and coping flexibility. COR theory therefore provides a useful basis for explaining how resilience-related resources may weaken stress-, anxiety-, and fatigue-related pathways.

Collegiate tennis provides a useful illustrative context for examining these processes because it requires sustained concentration, rapid tactical decision-making, individualized accountability, and repeated emotional recovery during competition. Tennis players must regulate their thoughts and emotions between points, respond to momentum shifts, maintain tactical flexibility, and recover from errors without continuous in-match support. These features make tennis a particularly visible example of how cognitive effort, competitive anxiety, and self-regulation may contribute to mental fatigue. However, the present review does not treat tennis as the exclusive focus of the framework. Instead, tennis is used as an applied example within a broader behavioral science discussion of collegiate athletes, whose academic–athletic dual roles create repeated conditions for stress appraisal, anxiety response, and cognitive–emotional depletion.

Despite growing interest in athlete mental health and mental fatigue, the existing literature remains fragmented. Studies often examine perceived stress, competitive anxiety, psychological resilience, and mental fatigue separately rather than as linked processes. In addition, mental fatigue has been measured inconsistently across studies, using self-report symptoms, perceived effort, cognitive tasks, physiological markers, or sport-performance indicators. This makes it difficult to compare findings across populations and to determine how stress and anxiety contribute to fatigue-related impairment over time. There is also limited evidence from non-Western collegiate athlete populations, where educational expectations, cultural norms, institutional support, and help-seeking patterns may shape how athletes experience and regulate psychological demands. These gaps highlight the need for more integrated pathway models, more consistent measurement strategies, and more context-sensitive research on collegiate athlete mental fatigue.

Therefore, this structured narrative review examines mental fatigue in collegiate athletes through the linked roles of perceived stress, competitive anxiety, and psychological resilience. It addresses three guiding questions: first, how mental fatigue can be conceptualized as a cognitive–emotional and psychobiological outcome in collegiate athletes; second, how perceived stress and competitive anxiety may contribute to fatigue-related impairment; and third, how psychological resilience may function as a protective regulatory resource. These questions are grounded in the Transactional Model of Stress and Coping, which emphasizes appraisal and coping resources in the stress process (Lazarus & Folkman, 1984), and Conservation of Resources theory, which explains how psychological strain may emerge when valued resources are threatened, depleted, or insufficiently restored (Hobfoll, 1989).

The aim of this review is to organize dispersed literature into a testable conceptual framework rather than to provide empirical validation of a causal pathway. Specifically, this review clarifies how mental fatigue can be positioned as a cognitive–emotional and psychobiological outcome, how perceived stress and competitive anxiety can be distinguished by their appraisal-based and proximal emotional roles, and how resilience, recovery, and contextual support may function as regulatory conditions. In this sense, the framework extends existing stress-coping and resilience perspectives by applying them to the dual academic–athletic environment of collegiate athletes.

Tennis and Chinese/non-Western collegiate sport contexts are used to illustrate how the proposed mechanisms may appear in specific settings, not to claim that the framework has been proven in those populations. They therefore remain examples of broader conceptual application rather than stronger evidential foundations.

## 2. Review Approach

This article adopts a structured narrative review approach to synthesize conceptual, theoretical, and empirical scholarship on mental fatigue in collegiate athletes, with particular attention to perceived stress, competitive anxiety, and psychological resilience. A structured narrative design was selected because the relevant literature is conceptually broad, methodologically heterogeneous, and distributed across several overlapping fields, including behavioral science, sport psychology, athlete mental health, stress research, cognitive fatigue, self-regulation, and performance psychology. Unlike a systematic review or meta-analysis, which aims to answer a narrowly defined question through exhaustive database searching, formal screening, risk-of-bias assessment, and quantitative aggregation, the present review aims to clarify concepts, compare theoretical perspectives, identify recurring mechanisms, and develop an integrative framework for future research (Baumeister & Leary, 1997; Grant & Booth, 2009; Snyder, 2019). Therefore, this review should be understood as a structured and transparent conceptual synthesis rather than a PRISMA-guided systematic review.

This review was organized around four interrelated constructs: mental fatigue, perceived stress, competitive anxiety, and psychological resilience. These constructs were selected because they represent theoretically meaningful components of a proposed stress–anxiety–fatigue pathway in collegiate athletes. Mental fatigue was treated as the central cognitive–emotional and psychobiological outcome. Perceived stress was positioned as an upstream appraisal-based condition reflecting how athletes interpret academic, competitive, and psychosocial demands. Competitive anxiety was considered a proximal emotional and attentional mechanism through which evaluative pressure may disrupt concentration, decision-making, and emotional regulation. Psychological resilience was examined as a dynamic regulatory resource that may support coping, recovery, and functional stability under pressure. This organization was guided by the Transactional Model of Stress and Coping, which emphasizes appraisal and coping resources in the stress process (Lazarus & Folkman, 1984), and Conservation of Resources theory, which explains how psychological strain may develop when valued resources are threatened, depleted, or insufficiently restored (Hobfoll, 1989).

Relevant literature was identified through structured searches of Scopus, Web of Science, PubMed, and Google Scholar. The searches focused on English-language peer-reviewed literature published up to February 2026. The search process combined construct-based, population-based, and context-based terms. Core search strings included combinations such as “mental fatigue” AND “athlete*”, “mental fatigue” AND “sport performance”, “cognitive fatigue” AND “athlete*”, “student-athlete*” OR “collegiate athlete*” AND “stress”, “perceived stress” AND “athlete*”, “stress appraisal” AND “sport”, “competitive anxiety” OR “sport anxiety” AND “athlete*”, “psychological resilience” AND “sport”, “resilience” AND “student-athlete*”, and “mental fatigue” AND “self-regulation” AND “performance”. Because tennis and Chinese/non-Western collegiate sport contexts were used as illustrative applications, additional searches combined terms such as “tennis”, “racket sport”, “individual sport”, “Chinese collegiate athletes”, “non-Western athletes”, “athlete mental health”, “help-seeking”, “academic-athletic role conflict”, “sleep”, “recovery”, and “burnout”. Backward citation tracking was also used to identify foundational theoretical works and frequently cited empirical studies relevant to stress, coping, resilience, competitive anxiety, mental fatigue, and athlete mental health.

The literature selection followed an eligibility logic based on conceptual relevance rather than formal PRISMA screening. Sources were considered relevant when they contributed directly to at least one of the following aims: defining or conceptualizing mental fatigue; explaining how perceived stress or stress appraisal may contribute to cognitive–emotional strain; clarifying the role of competitive anxiety in attentional control, emotional regulation, or performance disruption; examining psychological resilience as a protective or regulatory process; identifying recovery, sleep, burnout, or academic–athletic role conflict as related contextual factors; or highlighting measurement and cultural gaps in collegiate athlete research. Priority was given to peer-reviewed empirical studies, theoretical papers, systematic reviews, meta-analyses, and conceptual reviews. Foundational theoretical sources were included when they provided essential conceptual grounding for the framework. Studies were not excluded solely because they were not tennis-specific, because dedicated research on mental fatigue among collegiate tennis players remains limited. Instead, evidence from broader collegiate athlete samples, athlete mental health research, individual sports, racket sports, and cognitively demanding performance contexts was included when it clarified mechanisms relevant to the proposed framework.

Sources were not prioritized when they focused primarily on unrelated clinical, occupational, or non-athlete populations without clear relevance to stress appraisal, competitive anxiety, resilience, recovery, or mental fatigue in sport or performance contexts. Non-peer-reviewed sources were generally avoided unless they provided necessary contextual information. Because this review is narrative rather than systematic, no formal PRISMA flow diagram, risk-of-bias assessment, or meta-analytic procedure was applied. This limitation is acknowledged because narrative reviews are more vulnerable to selection bias than systematic reviews. To reduce this risk, the present review used a transparent construct-based search strategy, explicit eligibility logic, thematic grouping of evidence, and critical distinction between direct empirical support, evidence from adjacent athlete populations, and theoretical inference.

Relevance, quality, and representativeness were judged through an interpretive screening process consistent with the aims of a structured narrative review. Relevance was assessed according to whether a source directly clarified one of the four central constructs, explained relationships among stress appraisal, competitive anxiety, resilience, recovery, and mental fatigue, or provided contextual insight into collegiate athlete well-being. Quality was considered in terms of peer-reviewed status, conceptual clarity, methodological transparency, and contribution to established theoretical or empirical debates. Representativeness was addressed by including foundational theoretical works, recent reviews, empirical studies from athlete and student–athlete populations, and selected adjacent evidence from cognitively demanding sport or performance contexts. These judgments were not used as a formal risk-of-bias scoring procedure, but they helped ensure that the synthesis was conceptually balanced and not based only on isolated or highly selective findings.

The synthesis followed an interpretive and thematic procedure. After relevant literature was identified, sources were grouped according to their main contribution to this review: conceptual definitions of mental fatigue; stress appraisal and resource depletion; competitive anxiety and attentional regulation; resilience-related coping and recovery; psychobiological and behavioral mechanisms of mental fatigue; measurement heterogeneity; academic–athletic role conflict; sleep and recovery; cultural and institutional context; and practical implications for athlete support. These categories were then compared to identify convergences, inconsistencies, limitations, and unresolved questions in the literature. The purpose of this synthesis was not to claim causal validation of the proposed pathway, but to clarify how existing evidence and theory can be organized into a testable behavioral science framework for collegiate athlete mental fatigue.

To improve transparency, Table 1 summarizes the search strategy, eligibility logic, and synthesis procedure used in this review. In addition, because the proposed framework includes both empirically supported relationships and theoretically inferred pathways, a separate evidence-status table is provided later in this manuscript to distinguish direct evidence, adjacent evidence, and future hypotheses. This distinction is important because the framework is intended to guide future empirical testing rather than to represent a validated causal model.

**Figure 1:**
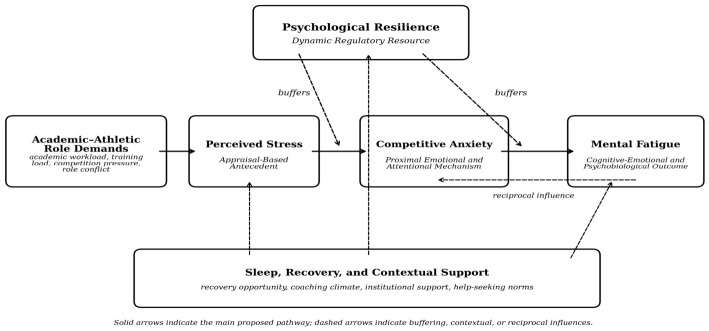
Revised conceptual heuristic framework linking academic–athletic demands, perceived stress, competitive anxiety, psychological resilience, recovery, contextual support, and mental fatigue in collegiate athletes. Note. The framework illustrates a theoretically informed pathway in which academic–athletic role demands may contribute to perceived stress, which may increase vulnerability to competitive anxiety and subsequent mental fatigue. Psychological resilience is positioned as a dynamic regulatory resource that may buffer the translation of perceived stress into competitive anxiety and the progression from competitive anxiety to fatigue-related impairment. Sleep, recovery opportunities, contextual support, coaching climate, institutional resources, and help-seeking norms are included as contextual and restorative factors that may influence resilience development and fatigue vulnerability. The dashed reciprocal pathway from mental fatigue to competitive anxiety indicates that fatigue may also weaken attentional control, emotional stability, and confidence, thereby increasing later anxiety. The model is intended as a conceptual heuristic framework for future empirical testing rather than a validated causal model. Tennis and Chinese/non-Western collegiate sport contexts are treated as illustrative applications rather than exclusive empirical targets.

The proposed behavioral mechanism framework presented in Figure 1 summarizes the integrative perspective developed in this review. The figure should be interpreted as a conceptual heuristic framework rather than a validated empirical model. It organizes existing theoretical and empirical literature by positioning perceived stress as an upstream appraisal-based condition, competitive anxiety as a proximal emotional and attentional mechanism, mental fatigue as a downstream cognitive–emotional and psychobiological outcome, and psychological resilience as a regulatory resource that may buffer fatigue-related vulnerability. The framework is intended to guide interpretation and future hypothesis development while recognizing that the proposed relationships may be influenced by recovery processes, academic–athletic role demands, institutional support, and cultural context.

### Evidence Status of the Proposed Framework

The framework shown in Figure 1 provides a conceptual roadmap for this review, but the proposed relationships should not be interpreted as equally established or as a fully validated causal sequence. To clarify how the framework should be read, Table 2 maps each major pathway or construct relationship onto its current evidence status. This evidence-status summary is included before the main conceptual sections so that the following discussion can distinguish direct empirical support, adjacent evidence from broader athlete or performance populations, and theoretical propositions that require future testing.

This evidence-status mapping provides the basis for the following sections, which first conceptualize mental fatigue as the central downstream outcome of the framework and then examine perceived stress, competitive anxiety, resilience-related regulation, contextual influences, measurement issues, and practical implications in greater detail.

## 3. Conceptualizing Mental Fatigue as a Cognitive–Emotional and Psychobiological Outcome

Mental fatigue has increasingly been recognized as a distinct cognitive–emotional and psychobiological state with important implications for human performance, self-regulation, and well-being. It is commonly understood as a state that arises after prolonged cognitive effort and is associated with reduced motivation, impaired attentional control, slower decision-making, increased perceived effort, and diminished capacity to sustain goal-directed behavior (Marcora et al., 2009; Van Cutsem et al., 2017; Pageaux & Lepers, 2018). From a behavioral science perspective, mental fatigue should not be reduced to simple tiredness or lack of effort. Rather, it reflects a disruption in the interaction between cognitive control, motivational regulation, affective stability, and perceived task demands. This distinction is especially important in collegiate athlete populations, where individuals may remain physically capable while still experiencing difficulty sustaining concentration, emotional control, and effective decision-making under pressure.

Mental fatigue also needs to be distinguished from related but non-identical constructs such as physical fatigue, general tiredness, burnout, and overtraining. Physical fatigue is usually associated with muscular exertion, metabolic disturbance, or physiological recovery demands, whereas mental fatigue is more closely linked to prior cognitive effort and reduced efficiency in sustained goal-directed performance (Van Cutsem et al., 2017; Pageaux & Lepers, 2018). General tiredness may be temporary and nonspecific, while mental fatigue is more directly connected to cognitive load, attentional depletion, and increased perceived effort. Burnout, by contrast, refers to a broader syndrome involving emotional and physical exhaustion, reduced sense of accomplishment, and devaluation of sport participation. Overtraining is typically discussed in relation to excessive training load and maladaptive physiological recovery. Although these conditions may overlap in real athletic environments, mental fatigue is conceptually narrower and more immediately linked to cognitive–emotional strain and self-regulatory disruption.

In collegiate athletes, mental fatigue may develop through the repeated accumulation of academic, competitive, and psychosocial demands. Student–athletes are required to shift between learning environments, training sessions, competition preparation, travel schedules, social expectations, and performance evaluation. These repeated transitions require sustained attention, working memory, emotional regulation, and coping effort. Over time, these demands may reduce the athlete’s capacity to maintain concentration, regulate frustration, and make effective decisions, particularly when recovery opportunities are limited. This interpretation is consistent with dual-career research showing that academic and athletic identities jointly influence athlete well-being and performance outcomes (Stambulova & Wylleman, 2019; van Rens et al., 2019). It is also consistent with student–athlete mental health research emphasizing that academic and sport demands can interact to increase psychological strain (Lu et al., 2012; Moreland et al., 2018; Kegelaers et al., 2024).

A key behavioral feature of mental fatigue is that it often changes the subjective cost of effort. Experimental work has shown that mentally fatigued individuals may report greater perceived effort even when major physiological indicators remain relatively stable (Marcora et al., 2009). This finding is important because it suggests that performance impairment under mental fatigue is not always caused by a direct loss of physical capacity. Instead, athletes may experience tasks as more demanding, less rewarding, or harder to sustain. In competitive settings, this may reduce persistence, weaken motivation, and make self-regulation more difficult. For collegiate athletes, this subjective increase in effort may be especially relevant because they frequently perform under conditions of cumulative cognitive load, academic pressure, and emotional evaluation.

Mental fatigue may also impair performance through disruptions in executive functioning. Executive functions such as attentional control, response inhibition, working memory, cognitive flexibility, and decision-making are central to successful athletic performance, especially in sports requiring rapid information processing and continuous adjustment. When mental fatigue reduces the efficiency of these functions, athletes may become more distractible, less able to inhibit impulsive responses, slower in adapting to changing conditions, and more vulnerable to negative self-talk or intrusive thoughts. Reviews of mental fatigue in sport suggest that such impairments can influence physical performance, skilled execution, and sport-specific psychomotor functioning (Habay et al., 2021; Sun et al., 2021). Therefore, mental fatigue should be understood not only as a subjective state but also as a condition that may affect observable performance through cognitive and behavioral pathways.

Collegiate tennis offers a useful applied example of these processes because it requires sustained concentration, rapid tactical decision-making, individualized accountability, and emotional recovery across repeated points. Tennis players must anticipate opponents’ intentions, select shots under time pressure, regulate emotional responses after errors, maintain tactical plans, and adapt to shifting score conditions. These demands rely heavily on attention, working memory, inhibition, and self-regulation. Evidence from racket-sport and precision-performance contexts suggests that mental fatigue can reduce aspects of skilled performance such as speed, accuracy, and control (Le Mansec et al., 2018; Sun et al., 2021). However, in the present review, tennis is treated as an illustrative context rather than the exclusive focus. The broader point is that collegiate athletes in cognitively demanding sports may be particularly vulnerable to mental fatigue when academic pressure, competitive anxiety, and insufficient recovery occur together.

Mental fatigue is therefore best conceptualized as a downstream outcome of sustained cognitive load, emotional strain, and resource depletion. This view aligns with Conservation of Resources theory, which proposes that individuals seek to protect and replenish valued resources such as attention, energy, confidence, emotional stability, and coping capacity (Hobfoll, 1989). When collegiate athletes repeatedly face academic and competitive demands without sufficient recovery, these resources may become depleted, increasing vulnerability to fatigue-related impairment. Mental fatigue may also be shaped by stress appraisal, as suggested by the Transactional Model of Stress and Coping, because athletes’ interpretations of demands as threatening, uncontrollable, or exceeding coping capacity may intensify psychological strain (Lazarus & Folkman, 1984). This theoretical framing supports the central argument of the present review: mental fatigue among collegiate athletes is not merely an isolated symptom, but a cognitive–emotional and psychobiological outcome embedded within broader processes of stress appraisal, competitive anxiety, resilience-related regulation, and recovery.

## 4. Perceived Stress as an Upstream Appraisal-Based Antecedent of Mental Fatigue

Perceived stress is an important upstream factor in understanding mental fatigue because it reflects how individuals evaluate the demands placed upon them rather than the objective presence of demands alone. From the perspective of the Transactional Model of Stress and Coping, stress emerges through a dynamic appraisal process in which individuals evaluate whether environmental demands are threatening, harmful, challenging, or manageable, and whether they possess sufficient coping resources to respond effectively (Lazarus & Folkman, 1984). This appraisal-based understanding is especially relevant for collegiate athletes, who are exposed to overlapping academic, competitive, interpersonal, and institutional demands. In such contexts, mental fatigue may not arise only from direct cognitive effort during training or competition, but also from repeated appraisals that ongoing demands are excessive, unpredictable, or difficult to control.

In collegiate sport, perceived stress may originate from multiple sources, including academic deadlines, examination pressure, training load, travel schedules, coach expectations, selection uncertainty, scholarship concerns, injury risk, interpersonal evaluation, and future career pressure. These stressors often do not occur separately. Instead, they accumulate across the athlete’s daily life and may create a continuous need for self-monitoring, emotional control, and coping effort. Student–athletes must frequently shift between academic concentration and athletic preparation, leaving limited time for psychological recovery. Previous research on college student–athletes has emphasized that sport-related and non-sport-related stressors can interact to influence psychological well-being, coping, and performance outcomes (Lu et al., 2012; Contreras et al., 2023; Kegelaers et al., 2024). Therefore, perceived stress should be understood as a central psychological context through which athletes interpret and respond to cumulative demands.

A key reason perceived stress may contribute to mental fatigue is that stress appraisal consumes cognitive and emotional resources. When athletes repeatedly perceive demands as threatening or exceeding their coping capacity, they may engage in sustained vigilance, worry, rumination, self-evaluation, and anticipatory regulation. These processes require attention, working memory, emotional inhibition, and motivational effort. Over time, such repeated regulatory activity may increase cognitive load and reduce the resources available for concentration, decision-making, and emotional stability. Research outside sport has also linked elevated perceived stress with exhaustion, anxiety, and depressive symptoms, supporting the view that perceived stress is not merely a subjective inconvenience but a broader vulnerability factor for psychological depletion (Wiegner et al., 2015). In athlete populations, this suggests that persistent perceived stress may increase susceptibility to fatigue-related impairment even before obvious physical or technical decline is visible.

Conservation of Resources theory provides a useful framework for explaining how perceived stress may gradually develop into mental fatigue (Hobfoll, 1989). According to this theory, individuals seek to acquire, protect, and restore valued resources, including energy, time, attention, confidence, emotional stability, and social support. Stress becomes harmful when these resources are threatened, lost, or insufficiently replenished. For collegiate athletes, academic workload, competitive pressure, and institutional expectations may repeatedly consume psychological resources while recovery opportunities remain limited. When this imbalance persists, athletes may enter a resource-depletion cycle in which the effort required to manage stress reduces the resources needed for future coping. Mental fatigue can therefore be understood as one possible downstream outcome of prolonged resource strain.

This resource-based interpretation is consistent with theoretical perspectives that use both the Transactional Model of Stress and Coping and Conservation of Resources theory to explain relationships among perceived stress, competitive anxiety, mental fatigue, and psychological resilience in athlete populations. In the present review, however, perceived stress is not treated as a statistical predictor within an empirical model. Instead, it is discussed as an appraisal-based antecedent that may initiate or intensify a broader cognitive–emotional pathway. This distinction allows this review to remain conceptually focused while avoiding the structure of an empirical hypothesis-testing study.

Perceived stress may also contribute to mental fatigue indirectly by increasing competitive anxiety. Athletes who appraise academic or sport-related demands as difficult to control may become more vulnerable to worry, self-doubt, and heightened arousal in evaluative performance situations. Competitive anxiety can then increase the cognitive cost of maintaining attention, regulating emotions, and making effective decisions under pressure. In this way, perceived stress may function as a broader background condition, while competitive anxiety may operate as a more immediate mechanism through which stress becomes fatigue-related impairment. This logic aligns with broader stress–fatigue frameworks in which competitive anxiety may help explain how perceived stress contributes to mental fatigue.

The relationship between perceived stress and mental fatigue may be particularly visible in cognitively demanding individual sports, such as tennis. Tennis players must often manage performance responsibility without continuous in-match support, regulate emotional responses after errors, and maintain tactical flexibility across changing score conditions. When perceived stress is already elevated because of academic obligations, selection pressure, or personal expectations, these competitive demands may become more cognitively costly. The athlete may experience stronger perceived effort, reduced attentional stability, and greater difficulty recovering emotionally between points or matches. However, tennis should be understood here as an illustrative example rather than the exclusive focus of the framework. The broader argument is that collegiate athletes in high-demand environments may become vulnerable to mental fatigue when repeated stress appraisal is combined with insufficient recovery and sustained self-regulatory demand.

Importantly, perceived stress does not necessarily impair performance or well-being in a uniform way. Some athletes may interpret demanding situations as challenges that can be managed, while others may interpret similar situations as threats that exceed their coping capacity. This difference highlights the importance of resilience, coping resources, and contextual support. Athletes with stronger psychological resources may be better able to reappraise demands, regulate emotional responses, and recover from strain before it develops into sustained mental fatigue. For this reason, perceived stress should not be viewed as a direct and deterministic cause of mental fatigue. Rather, it is best understood as an upstream appraisal-based condition whose effects may depend on emotional mechanisms, coping processes, and protective resources.

Overall, perceived stress provides a key starting point for understanding mental fatigue in collegiate athletes. It connects environmental demands with subjective appraisal, resource depletion, emotional regulation, and fatigue vulnerability. Within the framework proposed in this review, perceived stress is positioned as an upstream condition that may increase the likelihood of competitive anxiety and cognitive–emotional depletion. This interpretation provides a foundation for the Section 5, which examines competitive anxiety as a more proximal mechanism through which evaluative pressure may contribute to fatigue-related impairment.

## 5. Competitive Anxiety as a Proximal Emotional Mechanism in Fatigue-Related Impairment

Competitive anxiety is a central emotional mechanism in understanding how broader stress appraisals may develop into fatigue-related impairment among collegiate athletes. Whereas perceived stress reflects a broader appraisal of ongoing academic, athletic, and psychosocial demands, competitive anxiety is more closely tied to evaluative performance situations. It is commonly understood as a psychological and psychophysiological state involving worry, apprehension, tension, heightened arousal, and concerns about possible failure or negative evaluation (Woodman & Hardy, 2003). From a behavioral science perspective, competitive anxiety is important because it emerges at moments when athletes must preserve attentional control, regulate emotions, and make effective decisions under pressure. In this sense, competitive anxiety may serve as a proximal emotional pathway through which general stress becomes immediate cognitive–emotional strain.

Competitive anxiety is usually discussed through both cognitive and somatic dimensions. Cognitive anxiety includes worry, intrusive thoughts, self-doubt, fear of failure, and concerns about performance consequences. Somatic anxiety refers to bodily activation such as muscle tension, increased heart rate, sweating, and physical unease. Both dimensions may contribute to fatigue-related impairment, although they may do so through different pathways. Cognitive anxiety can consume attentional resources, increase rumination, and interfere with working memory and decision-making. Somatic anxiety may disrupt bodily comfort, movement rhythm, and perceived control. When these responses occur repeatedly across training and competition, athletes may need to invest additional mental effort simply to maintain focus and regulate emotional arousal. Over time, this increased regulatory demand may contribute to mental fatigue.

The attentional consequences of competitive anxiety are particularly important. Athletes experiencing high anxiety may become more vulnerable to threat-related cues, negative self-talk, and intrusive thoughts about mistakes or outcomes. Instead of allocating attention efficiently to task-relevant information, they may direct attention toward possible failure, external judgment, or internal discomfort. This shift can reduce attentional stability and increase the subjective effort required to perform. Research on anxiety and sport performance has shown that cognitive anxiety and self-confidence are strongly related to performance outcomes, suggesting that anxiety can influence performance through both attentional and motivational channels (Woodman & Hardy, 2003). For collegiate athletes, these effects may be intensified when competitive pressure is combined with academic workload and limited recovery time.

Competitive anxiety may also increase mental fatigue by raising the cost of self-regulation. In evaluative situations, athletes must not only execute skills but also regulate emotional reactions, inhibit distractions, manage bodily arousal, and maintain confidence after mistakes. These processes require continuous self-control. When anxiety is elevated, emotional regulation becomes more effortful, and the athlete may experience greater difficulty sustaining composure and decision quality. This is consistent with the broader mental fatigue literature, which suggests that performance impairment often occurs not because the athlete becomes immediately physiologically incapable, but because cognitive control, motivation, and effort regulation become progressively harder to sustain under mental load (Marcora et al., 2009; Pageaux & Lepers, 2018; Meeusen et al., 2021).

The relationship between competitive anxiety and mental fatigue is also linked to perfectionism and responses to failure. Athletes who react strongly to mistakes may experience higher levels of worry, frustration, and self-evaluation during competition. Research on perfectionism in athletes suggests that negative reactions to imperfection can intensify competitive anxiety and make evaluative pressure more difficult to regulate (Stoeber et al., 2007). Broader evidence on anxiety in athletes also indicates that anxiety is shaped by performance expectations, fear of failure, social evaluation, and perceived consequences of poor performance (Rice et al., 2019). These findings suggest that competitive anxiety may not only be a temporary emotional state, but also a mechanism that increases the psychological cost of performance, especially when athletes repeatedly interpret mistakes as threatening or personally significant.

In cognitively demanding individual sports, competitive anxiety may be especially visible because athletes often carry direct responsibility for performance outcomes. Tennis provides a useful applied example. During a match, players must make rapid tactical decisions, regulate emotional responses between points, recover from errors, and adjust to changing score conditions. When competitive anxiety is high, these demands may become more cognitively expensive. A player may hesitate before action, overthink tactical choices, struggle to reset after mistakes, or become overly reactive to momentum shifts. These anxiety-related processes can gradually reduce attentional efficiency and increase perceived effort, thereby contributing to mental fatigue. However, tennis is used here as an illustrative context rather than the exclusive focus; similar mechanisms may occur in other collegiate sports that require sustained attention, emotional control, and rapid decision-making.

Competitive anxiety may also help explain why perceived stress does not always translate directly into mental fatigue. Perceived stress may create a general background of psychological strain, but competitive anxiety may be the more immediate emotional state that transforms this strain into fatigue-related impairment during performance. For example, an athlete who appraises academic workload, coach expectations, or selection pressure as overwhelming may enter competition already psychologically burdened. Once evaluative pressure begins, competitive anxiety may intensify worry, self-monitoring, and physiological arousal, increasing the cognitive effort required to maintain performance. In this way, competitive anxiety can be understood as a proximal mechanism linking broader stress appraisal to mental fatigue.

This interpretation is consistent with evidence suggesting that organizational and performance-related stressors are associated with competitive anxiety and burnout in athletes, and that psychological resilience may moderate these relationships (Wu et al., 2022). Although burnout and mental fatigue are not identical constructs, this evidence supports the broader idea that anxiety-related responses can connect environmental stressors with psychological depletion. In the present review, competitive anxiety is therefore not treated as a secondary or incidental feature of sport participation. Rather, it is positioned as a central emotional mechanism through which stress may disrupt attention, self-regulation, and recovery, thereby increasing vulnerability to mental fatigue.

At the same time, the effect of competitive anxiety on fatigue-related impairment is unlikely to be uniform across athletes. Some athletes may experience anxiety as debilitating, while others may interpret arousal as manageable or even facilitative depending on their coping resources, confidence, prior experience, and resilience. This distinction is important because it prevents competitive anxiety from being understood as automatically harmful in every situation. Instead, its impact depends on how athletes appraise anxiety, regulate emotional responses, and access protective resources. Psychological resilience may therefore play an important role in weakening the pathway from competitive anxiety to mental fatigue by supporting emotional recovery, coping flexibility, and functional stability under pressure.

Overall, competitive anxiety represents a proximal emotional mechanism in the stress–fatigue pathway. It may increase mental fatigue by consuming attentional resources, intensifying self-monitoring, disrupting decision-making, increasing perceived effort, and raising the regulatory cost of performance. Within the framework proposed in this review, perceived stress is positioned as an upstream appraisal-based condition, competitive anxiety as a more immediate emotional and attentional mechanism, and mental fatigue as a downstream cognitive–emotional and psychobiological outcome. However, this pathway should not be interpreted as strictly linear or one-directional. Mental fatigue may also feed back into competitive anxiety by weakening attentional control, reducing emotional stability, increasing perceived effort, and undermining confidence during performance. A fatigued athlete may therefore become more vulnerable to worry, hesitation, negative self-evaluation, and threat-focused attention in later competitive situations.

This reciprocal interpretation is important because it prevents the proposed framework from oversimplifying the relationship between anxiety and fatigue. In applied collegiate sport settings, stress, anxiety, and fatigue are likely to unfold through repeated cycles rather than isolated events. Academic workload, training demands, competition pressure, sleep disruption, and insufficient recovery may accumulate across days or weeks, creating conditions in which competitive anxiety contributes to fatigue and fatigue subsequently increases anxiety vulnerability. Future research should therefore test both forward and reciprocal pathways using longitudinal, multi-wave, or daily-diary designs. This interpretation provides the foundation for the Section 6, which examines the psychobiological and behavioral mechanisms through which mental fatigue affects performance regulation and athlete functioning.

## 6. Psychobiological and Behavioral Mechanisms of Mental Fatigue

Mental fatigue influences athlete functioning through a combination of psychobiological, cognitive, emotional, and behavioral mechanisms. A central insight from the mental fatigue literature is that performance impairment does not necessarily occur because the body becomes immediately incapable of action. Rather, impairment often emerges because cognitive control, effort regulation, motivational stability, and behavioral consistency become progressively harder to sustain under prolonged mental load (Marcora et al., 2009; Pageaux & Lepers, 2018; Meeusen et al., 2021). From a behavioral science perspective, mental fatigue should therefore be understood as a condition that alters how athletes experience, regulate, and allocate effort across demanding tasks. The athlete may remain physically prepared, but performance becomes more difficult to maintain because attention is less stable, perceived effort is higher, and emotional regulation requires greater self-control.

One important mechanism involves executive functioning. Mental fatigue may reduce the efficiency of attentional control, response inhibition, working memory, cognitive flexibility, and decision-making. These functions are essential for athletes who must process rapidly changing information, select appropriate responses, inhibit impulsive actions, and adjust behavior under pressure. When executive control is weakened, athletes may become slower in decision-making, less able to filter irrelevant information, and more likely to rely on automatic or less adaptive responses. Reviews of mental fatigue in sport suggest that fatigue-related impairment is particularly evident in tasks that require sustained attention, psychomotor control, and complex decision-making (Habay et al., 2021; Sun et al., 2021). This indicates that mental fatigue affects not only subjective experience but also the cognitive operations that support skilled and adaptive behavior.

A second mechanism concerns attentional vulnerability. Under mental fatigue, athletes may have greater difficulty maintaining task-relevant focus and resisting distraction. This vulnerability can become especially problematic in competitive environments, where athletes are exposed to score pressure, mistakes, social evaluation, coaching expectations, and changing performance conditions. Fatigued athletes may become more reactive to threat-related cues, more affected by negative self-talk, and less able to sustain attentional stability over time. This mechanism is closely connected to competitive anxiety, because anxiety can direct attention toward possible failure or external judgment, while mental fatigue reduces the capacity to regulate those attentional shifts. Together, anxiety and fatigue may create a cycle in which worry consumes cognitive resources, reduced control increases perceived difficulty, and rising effort further intensifies fatigue-related impairment.

A third mechanism involves perceived effort. One of the most widely discussed findings in mental fatigue research is that mentally fatigued individuals often report greater effort even when major physiological indicators remain relatively stable (Marcora et al., 2009). This suggests that mental fatigue changes the subjective cost of sustaining performance. Athletes may experience ordinary tasks as more demanding, less rewarding, or harder to continue. This increase in perceived effort is important because sport performance often depends not only on physical ability, but also on the willingness to persist, regulate frustration, and continue making effective decisions under pressure. When the subjective cost of effort rises, athletes may become less patient, less tactically disciplined, and less motivated to sustain high-quality performance over time (Pageaux & Lepers, 2018; Van Cutsem et al., 2017).

A fourth mechanism is emotional and self-regulatory instability. Mental fatigue can reduce the athlete’s capacity to regulate frustration, recover from mistakes, and maintain confidence during difficult performance episodes. Because self-regulation requires cognitive and emotional resources, fatigue may make it harder for athletes to inhibit impulsive reactions, reinterpret setbacks constructively, or return attention to task-relevant cues. In this way, mental fatigue may amplify the emotional cost of competition. Athletes may become more irritable, more sensitive to errors, and more vulnerable to confidence disruption. This mechanism helps explain why mental fatigue should not be viewed only as cognitive tiredness. It also involves affective regulation, motivational control, and behavioral persistence.

These mechanisms are particularly relevant in collegiate athlete populations because academic and athletic demands often interact. A student–athlete may enter training or competition after several hours of academic concentration, examination preparation, travel, or institutional obligations. Even before sport-specific performance begins, cognitive resources may already be partially depleted. When competitive pressure is added, the athlete must regulate attention, emotion, effort, and decision-making under conditions of reduced recovery. Dual-career research has emphasized that academic and athletic roles jointly shape well-being and performance, while student–athlete mental health research has shown that academic–athletic demands can influence psychological strain and help-seeking needs (Stambulova & Wylleman, 2019; van Rens et al., 2019; Moreland et al., 2018; Kegelaers et al., 2024). These findings support the view that mental fatigue in collegiate athletes is embedded within a broader behavioral and institutional context.

Collegiate tennis offers a useful applied example of how these mechanisms may operate. Tennis requires athletes to sustain concentration across repeated points, interpret opponents’ intentions, select tactical responses under time pressure, regulate emotions after errors, and adapt to changing match conditions. When mental fatigue is present, these demands may become more difficult because attentional control weakens, perceived effort rises, and emotional recovery becomes less efficient. Evidence from racket-sport and precision-performance contexts suggests that mentally fatiguing conditions can impair aspects of skilled performance such as ball speed, accuracy, and control (Le Mansec et al., 2018). However, tennis should be understood as one example rather than the sole focus of the framework. Similar mechanisms may apply across other cognitively demanding collegiate sports where athletes must sustain attention, regulate emotion, and make repeated decisions under evaluative pressure.

The psychobiological interpretation of mental fatigue also aligns with Conservation of Resources theory. If attention, energy, confidence, emotional stability, and coping capacity are treated as valued psychological resources, then mental fatigue can be understood as a condition emerging when these resources are repeatedly consumed without sufficient restoration (Hobfoll, 1989). In this sense, mental fatigue reflects more than immediate cognitive tiredness. It represents a broader resource-regulation problem in which the athlete’s ability to sustain effort, maintain control, and recover emotionally becomes compromised. The Transactional Model of Stress and Coping further suggests that appraisal processes shape this pathway, because athletes who interpret demands as threatening or exceeding coping capacity may experience greater regulatory burden and faster depletion (Lazarus & Folkman, 1984).

Overall, mental fatigue affects collegiate athletes through multiple interacting mechanisms. It may weaken executive functioning, increase attentional vulnerability, raise perceived effort, disrupt emotional regulation, and reduce behavioral consistency. These mechanisms explain why athletes may appear physically prepared while still experiencing meaningful difficulties in concentration, decision-making, emotional control, and performance regulation. Within the framework proposed in this review, mental fatigue is therefore conceptualized as a downstream cognitive–emotional and psychobiological outcome that develops through the interaction of stress appraisal, competitive anxiety, resource depletion, and resilience-related regulation.

At the same time, mental fatigue should not be understood only as the final endpoint of a one-directional pathway. Once fatigue develops, it may feed back into the stress–anxiety process by weakening attentional control, increasing perceived effort, reducing confidence, and making emotional recovery more difficult. This feedback may increase vulnerability to later competitive anxiety, especially when academic workload, training demands, poor sleep, and insufficient recovery continue across repeated performance cycles. In this sense, mental fatigue may be both an outcome of stress and anxiety and a condition that increases later anxiety-related vulnerability. This reciprocal interpretation strengthens the framework by recognizing that collegiate athletes’ psychological responses unfold dynamically across academic, training, and competitive contexts rather than through a single linear sequence. The Section 7 examines psychological resilience as a protective regulatory resource that may buffer these fatigue-related vulnerabilities by supporting appraisal, coping flexibility, recovery, and functional stability under pressure.

## 7. Psychological Resilience as a Protective Regulatory Resource

Psychological resilience is a key construct for explaining why collegiate athletes exposed to similar stressors and competitive pressures may experience different levels of mental fatigue. Although all athletes encounter demanding academic, athletic, and psychosocial conditions, their responses to these demands are not uniform. Some athletes may experience persistent worry, emotional instability, and cognitive depletion, whereas others may recover more effectively and maintain functional performance under pressure. Resilience helps explain this variability because it reflects the capacity to adapt positively to adversity, recover from stress, and maintain psychological functioning in the face of challenge (Fletcher & Sarkar, 2012, 2013). From a behavioral science perspective, resilience should therefore be understood not merely as a stable personal trait, but as a protective regulatory resource that shapes appraisal, coping, emotional recovery, and sustained self-regulation.

Early resilience research often treated resilience as a relatively stable individual characteristic, but contemporary perspectives increasingly emphasize its dynamic and context-dependent nature. In sport psychology, resilience is commonly viewed as a process through which athletes interact with adversity, draw on personal and environmental resources, and develop adaptive responses over time (Fletcher & Sarkar, 2012; Sarkar & Fletcher, 2014). This process-oriented understanding is especially relevant for collegiate athletes because they are repeatedly exposed to both short-term pressures, such as competitions and examinations, and longer-term demands, such as identity development, academic progression, and athletic career uncertainty. Resilience in this context is not the absence of stress or anxiety; rather, it is the ability to regulate the consequences of stress and recover before temporary strain develops into more persistent fatigue-related impairment.

Resilience may operate protectively at several points within the stress–anxiety–fatigue pathway. At the appraisal stage, resilience may help athletes interpret demanding situations as manageable challenges rather than overwhelming threats. Athletes with stronger resilience-related resources may be less likely to view setbacks, competitive uncertainty, or academic pressure as uncontrollable or personally destructive. Resilience may also support secondary appraisal by strengthening perceived coping capacity, confidence, and the belief that effective responses are available. This interpretation is consistent with the Transactional Model of Stress and Coping, which emphasizes that stress responses depend not only on external demands but also on how individuals evaluate their coping resources (Lazarus & Folkman, 1984).

At the emotional regulation stage, resilience may reduce the intensity, duration, or performance cost of competitive anxiety. Competitive anxiety can increase worry, intrusive thoughts, bodily tension, self-monitoring, and fear of failure. These processes are mentally costly because they require athletes to invest additional effort in emotional regulation and attentional control. Resilient athletes may be better able to reinterpret arousal, use flexible coping strategies, maintain confidence after mistakes, and restore attentional focus in evaluative situations. Previous research has emphasized that resilient athletes often draw on adaptive coping, confidence, motivation, and social support to manage pressure and sustain performance functioning (Galli & Vealey, 2008; Fletcher & Sarkar, 2012; Sarkar & Fletcher, 2014). These resources may help prevent competitive anxiety from escalating into prolonged cognitive–emotional depletion.

At the recovery stage, resilience may support the restoration of cognitive and emotional resources after stress exposure. Mental fatigue is not influenced only by the intensity of demands, but also by whether athletes have sufficient opportunity and support to recover from those demands. According to Conservation of Resources theory, individuals seek to protect and replenish valued resources such as energy, attention, confidence, emotional stability, and social support (Hobfoll, 1989). When these resources are repeatedly threatened or depleted, fatigue-related impairment becomes more likely. Resilience may reduce this risk by supporting resource recovery, flexible coping, and adaptive re-engagement after setbacks. In collegiate sport, this may include recovering emotionally after poor performance, maintaining motivation during academic pressure, preserving confidence after competitive failure, and using available social or institutional support to prevent temporary strain from becoming sustained fatigue.

It is important, however, not to romanticize resilience as invulnerability. Resilient athletes still experience stress, anxiety, frustration, disappointment, and temporary fatigue. The difference is not that they are unaffected by adversity, but that they may be better able to regulate its psychological consequences. Treating resilience as invulnerability can be problematic because it may place excessive responsibility on individual athletes while ignoring environmental conditions that contribute to stress and fatigue. A more balanced interpretation recognizes that resilience develops through the interaction of personal resources, coping experiences, supportive relationships, coaching climates, and institutional structures (Galli & Vealey, 2008; Sarkar & Fletcher, 2014). This view is especially important in university sport systems, where athletes’ coping capacity may depend on academic flexibility, psychological support services, coach communication, and recovery opportunities.

Resilience is also closely related to coping, but the two constructs should not be treated as identical. Coping refers to the cognitive and behavioral strategies individuals use to manage specific demands, whereas resilience refers to the broader capacity to adapt, recover, and maintain functioning across adversity. Adaptive coping strategies, such as cognitive reappraisal, problem-solving, attentional refocusing, relaxation, and seeking social support, may contribute to resilience development over time. Conversely, stronger resilience may make athletes more likely to select flexible and adaptive coping strategies when stressors arise. Research on coping in sport has shown that coping responses can influence how athletes manage stress and maintain performance under pressure (Nicholls & Polman, 2007). Therefore, resilience may be understood as both supported by coping processes and expressed through coping behavior.

Within the framework proposed in this review, psychological resilience is positioned as a protective regulatory resource rather than as a simple personality variable. This distinction is important because it allows resilience to be linked to stress appraisal, emotional regulation, and fatigue recovery without reducing it to a fixed trait. Resilience may weaken the pathway from perceived stress to competitive anxiety by shaping how athletes appraise demands and evaluate coping resources. It may also weaken the pathway from competitive anxiety to mental fatigue by supporting emotional recovery, attentional refocusing, and confidence maintenance. In this sense, resilience contributes to the regulation of cognitive–emotional load before it develops into sustained mental fatigue.

Collegiate tennis offers a useful applied example of how resilience may function under pressure. Tennis players must often respond to mistakes without immediate team support, regulate emotions between points, and sustain tactical discipline across changing score conditions. A resilient player may be better able to recover after an error, reinterpret competitive pressure as manageable, and maintain attentional focus during momentum shifts. However, tennis should be treated as an illustrative context rather than the exclusive focus of the present framework. Similar resilience-related processes may operate across collegiate sports that require repeated self-regulation, emotional control, and recovery under evaluative pressure.

The practical significance of resilience lies in its developability. If resilience is understood as a dynamic regulatory resource, then it can be strengthened through psychological skill training, reflective coping, supportive coaching, confidence-building, mindfulness-based strategies, and structured recovery practices. Psychological skill training has been associated with enhanced athlete performance and well-being, particularly when it targets attention, confidence, emotional regulation, and coping under pressure (Birrer & Morgan, 2010; Foster & Chow, 2020; Golby & Wood, 2016). These approaches may help athletes reduce the likelihood that stress and anxiety develop into sustained mental fatigue. At the institutional level, resilience development may also depend on whether universities provide accessible psychological support, reasonable scheduling, and environments that normalize help-seeking.

Overall, psychological resilience provides a critical protective component in the behavioral science framework of mental fatigue. It helps explain why perceived stress and competitive anxiety do not affect all collegiate athletes in the same way. By shaping appraisal, supporting coping flexibility, strengthening emotional recovery, and preserving regulatory resources, resilience may reduce vulnerability to fatigue-related impairment. The Section 8 extends this discussion by considering how cultural, institutional, and non-Western collegiate athlete contexts may shape the experience and regulation of mental fatigue.

## 8. Contextual and Cultural Considerations in Collegiate Athlete Settings

Mental fatigue among collegiate athletes should not be understood as a purely individual or sport-specific phenomenon. Although cognitive load, competitive pressure, and self-regulatory demands are experienced at the individual level, these processes are shaped by broader cultural, educational, and institutional contexts. Athletes do not appraise stress, regulate anxiety, or recover from fatigue in a social vacuum. Their experiences are influenced by university expectations, family values, coaching climates, help-seeking norms, academic systems, and the availability of psychological support. For this reason, a behavioral science perspective on mental fatigue should consider not only internal psychological mechanisms but also the environments in which athletes study, train, compete, and recover.

This contextual perspective is especially important in non-Western collegiate athlete settings, where educational expectations and cultural norms may shape how athletes interpret pressure and respond to psychological strain. In many Asian higher education contexts, academic success is closely linked to family expectations, future mobility, social responsibility, and long-term career security. For student–athletes, this may create a dual burden in which academic performance and athletic achievement are both experienced as meaningful obligations. When athletes perceive these demands as difficult to balance, the resulting stress appraisal may increase emotional strain and reduce opportunities for psychological recovery. This interpretation is consistent with broader student–athlete research showing that academic and athletic demands may interact to increase stress, anxiety, and fatigue vulnerability.

The dual-role structure of collegiate sport is particularly relevant here. Student–athletes must repeatedly shift between academic identity and athletic identity, often within compressed schedules that require concentration, physical preparation, travel, and competition. Dual-career scholarship has emphasized that academic and athletic identities jointly shape well-being, adjustment, and performance outcomes (Stambulova & Wylleman, 2019; van Rens et al., 2019). When these roles are experienced as compatible, they may support motivation and personal development. However, when they are experienced as competing demands, they may contribute to role conflict, stress, and fatigue vulnerability. In this sense, mental fatigue may develop not only from sport participation itself, but also from repeated transitions between educational and athletic performance systems.

Cultural expectations may also influence how athletes express distress and seek support. In some contexts, athletes may be reluctant to disclose stress, anxiety, or fatigue because they fear appearing weak, disappointing coaches or family members, losing selection opportunities, or being judged by peers. Help-seeking may be further shaped by stigma, uncertainty about confidentiality, and limited access to sport psychology services. Reviews of collegiate athlete mental health have shown that barriers to help-seeking include stigma, lack of mental health literacy, limited service availability, and concerns about how disclosure may affect athletic identity or team status (Moreland et al., 2018; Cosh et al., 2024; Kegelaers et al., 2024). These barriers are important because unaddressed stress and anxiety may accumulate over time and contribute to sustained cognitive–emotional depletion.

Institutional conditions also matter. University athletes’ ability to recover from psychological strain depends partly on whether their environment provides reasonable training schedules, academic flexibility, accessible psychological services, supportive coaching, and recovery opportunities. If institutional systems prioritize performance outcomes while neglecting recovery and mental health support, athletes may be more vulnerable to resource depletion. Conservation of Resources theory is useful in this regard because it emphasizes that stress becomes harmful when valued resources are threatened, lost, or insufficiently restored (Hobfoll, 1989). In collegiate sport, such resources may include time, attention, emotional stability, confidence, social support, and access to professional help. When institutional structures repeatedly consume these resources without adequate replenishment, mental fatigue may become more likely.

The Chinese collegiate sport context provides a useful example of why culturally grounded approaches are needed. China’s higher education environment can place strong emphasis on academic achievement, future employability, family expectations, and institutional reputation. For collegiate athletes, these pressures may coexist with training demands, competition schedules, selection systems, and expectations for athletic progress. Research on collegiate tennis players has identified perceived stress, competitive anxiety, mental fatigue, and psychological resilience as key constructs for understanding athlete adaptation in this population. However, in the present review, China and tennis should be understood as illustrative contexts rather than as the only setting of interest.

Tennis remains a useful applied example because it combines individual accountability, repeated decision-making, emotional recovery, and limited in-match support. In such a context, athletes may internalize mistakes strongly and experience heightened self-evaluation during competition. When this performance structure is combined with academic pressure and cultural expectations, stress appraisal and competitive anxiety may become more cognitively costly. However, similar mechanisms may also occur in other individual or cognitively demanding sports where athletes must manage pressure largely through self-regulation. Therefore, tennis should be used to illustrate the framework, not to restrict its relevance.

A culturally sensitive perspective also affects how resilience should be understood. Resilience is often discussed as an individual capacity, but athletes’ ability to remain adaptive under pressure depends on more than personal toughness. It is shaped by social support, coaching relationships, institutional resources, cultural meanings of success and failure, and the acceptability of seeking psychological help. Treating resilience only as an individual trait risks overlooking the environmental conditions that produce stress and fatigue. A more balanced view understands resilience as a regulatory resource that develops through interactions between personal coping capacity and contextual support (Fletcher & Sarkar, 2013; Sarkar & Fletcher, 2014). This interpretation is especially important for collegiate athlete populations because universities can either strengthen or weaken athletes’ recovery resources.

Overall, contextual and cultural considerations are essential for understanding mental fatigue in collegiate athletes because they may shape both the meaning of demands and the availability of recovery resources. Stress appraisal, competitive anxiety, resilience-related regulation, and mental fatigue are not only individual psychological processes; they are also influenced by coaching relationships, academic flexibility, institutional support, help-seeking norms, and culturally shaped expectations about success, failure, endurance, and psychological disclosure. This means that the same academic–athletic demand may have different psychological consequences across university systems, sports, and cultural settings depending on how athletes interpret pressure and what support resources are available.

For this reason, the present review treats tennis and Chinese/non-Western collegiate sport contexts as illustrative applications rather than as restrictive boundaries of the framework. Their value is not that they represent all collegiate athletes, but that they make visible contextual factors that are often underexamined in broader sport psychology models. Tennis illustrates how individual accountability, repeated decision-making, and limited in-match support may intensify self-regulatory demands, while Chinese and other non-Western collegiate contexts illustrate how academic expectations, institutional structures, cultural norms, and help-seeking barriers may shape fatigue vulnerability. Future research should therefore test the proposed framework across different sports, university systems, and cultural contexts to determine which pathways are generalizable and which are context-specific. This contextual clarification strengthens the framework by positioning culture and institution as active moderators of the stress–anxiety–fatigue process rather than as background descriptions.

## 9. Measurement Issues, Conceptual Gaps, and Future Research Needs

Despite growing interest in mental fatigue, athlete mental health, and psychological resilience, the literature remains limited by conceptual inconsistency, measurement heterogeneity, and insufficient integration across stress, anxiety, fatigue, and resilience research. Mental fatigue is frequently discussed alongside related constructs such as burnout, general tiredness, emotional exhaustion, overtraining, and low motivation. Although these constructs may overlap in applied sport settings, they are not conceptually identical. Mental fatigue is more specifically linked to prolonged cognitive effort, reduced attentional efficiency, increased perceived effort, impaired executive functioning, and diminished self-regulatory capacity (Van Cutsem et al., 2017; Pageaux & Lepers, 2018). Without clearer conceptual boundaries, researchers may risk attributing fatigue-related impairment to general psychological distress or poor motivation rather than to cognitive–emotional depletion.

A major limitation concerns the measurement of mental fatigue. Across existing studies, mental fatigue has been assessed using self-report scales, perceived effort ratings, cognitive-performance tasks, psychomotor tests, physiological indicators, and sport-specific performance outcomes. Each approach captures a different aspect of the construct. Self-report measures may reflect subjective symptoms such as tiredness, reduced motivation, difficulty concentrating, and perceived cognitive strain. Cognitive tasks may assess attention, inhibition, working memory, or decision-making under fatigue conditions. Physiological indicators may provide indirect information about arousal or recovery status, while sport-performance measures may show how fatigue affects skilled execution. However, no single method has become dominant in sport and collegiate athlete research, making it difficult to compare findings across studies or establish consistent thresholds for fatigue-related impairment (Duignan et al., 2020; Habay et al., 2021; Sun et al., 2021).

This measurement problem is especially important because mental fatigue is both subjective and functional. An athlete may report high perceived mental fatigue even when objective physiological markers remain relatively stable, while another athlete may show impaired decision-making or attentional control before explicitly recognizing fatigue symptoms. Experimental research has shown that mental fatigue can increase perceived effort without necessarily producing parallel changes in major physiological indicators (Marcora et al., 2009). Therefore, relying on only one type of measure may produce an incomplete picture. Future research would benefit from multi-method designs that combine self-report assessment, behavioral indicators, cognitive tasks, and, where feasible, psychobiological measures. Such approaches would make it easier to distinguish mental fatigue from adjacent states and to identify when fatigue becomes meaningful for athlete functioning.

Measurement heterogeneity is not limited to mental fatigue alone. Perceived stress, competitive anxiety, and psychological resilience are also assessed through instruments that differ in time frame, dimensional structure, and contextual specificity. For example, perceived stress measures may capture general life stress, academic stress, or sport-related appraisal, while competitive anxiety measures may emphasize cognitive worry, somatic activation, self-confidence, or pre-competition state anxiety. Resilience measures may also differ in whether they treat resilience as a relatively stable trait, a coping-related capacity, or a dynamic adaptation process. These differences make it difficult to determine whether studies are examining the same psychological mechanisms or only related but non-equivalent constructs. For collegiate athlete research, this issue is especially important because stress, anxiety, resilience, and fatigue may fluctuate across academic semesters, training cycles, examination periods, injury recovery, and competition schedules.

Future research should therefore report measurement choices more transparently and align instruments with the theoretical role assigned to each construct. If perceived stress is treated as an appraisal-based antecedent, measures should capture athletes’ subjective evaluation of academic–athletic demands rather than only objective workload. If competitive anxiety is treated as a proximal emotional mechanism, assessment should be sensitive to performance context and timing, such as pre-competition or in-competition anxiety. If resilience is treated as a dynamic regulatory resource, studies should consider whether the measure captures coping flexibility, recovery capacity, contextual support, or stable individual differences. Greater attention to measurement timing, construct definition, and cultural validity would improve the comparability of findings and would support stronger empirical testing of the proposed stress–anxiety–fatigue framework.

A second gap concerns the limited integration of perceived stress, competitive anxiety, psychological resilience, and mental fatigue within a single explanatory framework. Many studies examine these constructs separately, focusing on direct associations between stress and well-being, anxiety and performance, or resilience and coping. However, fewer studies consider how these variables may operate as linked processes. From a behavioral science perspective, perceived stress may function as an upstream appraisal-based condition, competitive anxiety as a proximal emotional mechanism, psychological resilience as a protective regulatory resource, and mental fatigue as a downstream cognitive–emotional and psychobiological outcome. This integrative pathway is consistent with the theoretical logic of the Transactional Model of Stress and Coping and Conservation of Resources theory, both of which emphasize appraisal, coping resources, and depletion processes (Hobfoll, 1989; Lazarus & Folkman, 1984). These theories provide a useful conceptual basis for linking perceived stress, competitive anxiety, mental fatigue, and psychological resilience without requiring the present review to adopt an empirical SEM structure.

A third limitation is the shortage of longitudinal evidence. Much of the available literature relies on cross-sectional designs, laboratory fatigue protocols, or short-term performance tasks. These designs are useful for identifying associations or testing immediate fatigue effects, but they are less able to explain how mental fatigue develops across academic semesters, training cycles, examination periods, competition seasons, injury recovery, and transitions between sport and study. Collegiate athletes experience fatigue within repeated cycles of academic and athletic demands, meaning that fatigue vulnerability may fluctuate over time. Longitudinal studies are therefore needed to clarify whether perceived stress predicts later competitive anxiety, whether competitive anxiety contributes to accumulated fatigue, and whether resilience reduces fatigue vulnerability across time rather than only at a single measurement point.

A fourth gap concerns population specificity. Existing research on mental fatigue and athlete well-being often draws on elite athletes, team-sport athletes, general student samples, or mixed sport populations. Although these studies provide valuable insight, they may not fully capture the experiences of collegiate athletes who must balance academic progression and athletic performance. Student–athletes face dual-role pressures that differ from those of professional athletes and non-athlete students. They must manage performance expectations while also meeting educational requirements, institutional responsibilities, and identity-related demands. Reviews of student–athlete mental health and help-seeking emphasize the need for more context-sensitive research that accounts for academic–athletic role conflict, service access, stigma, and institutional support (Moreland et al., 2018; Cosh et al., 2024; Kegelaers et al., 2024).

A fifth limitation concerns cultural and institutional diversity. Much of the literature used to develop current models of athlete stress, anxiety, resilience, and fatigue has been generated in Western or elite sport contexts. However, athletes in non-Western collegiate systems may experience different academic expectations, family obligations, coaching structures, institutional resources, and help-seeking norms. These differences may influence how athletes appraise stress, express anxiety, access support, and recover from mental fatigue. For this reason, future research should include more culturally diverse collegiate athlete populations and avoid assuming that models developed in one context will automatically generalize to another. This is especially relevant for Chinese university sport settings, where academic expectations and athletic development may be shaped by distinctive social and institutional pressures.

Future research should also clarify the role of resilience without reducing it to a simple trait or universal solution. Resilience is often described as protective, but its protective function may depend on coping strategies, social support, coaching climate, institutional resources, and recovery opportunities. Studies should therefore examine resilience not only as an individual variable but also as a dynamic regulatory process embedded within broader contexts. This may include testing whether resilience changes across a season, whether resilience-based interventions reduce fatigue vulnerability, and whether institutional support strengthens athletes’ ability to recover from stress and anxiety. Such work would help avoid overly individualistic explanations that place responsibility for fatigue solely on athletes while ignoring the environments that create and sustain psychological demands.

In methodological terms, future studies should move beyond simple direct-effect models and test more explicit pathway frameworks. Potential models may examine whether perceived stress predicts mental fatigue directly or indirectly through competitive anxiety, whether psychological resilience moderates the pathway from perceived stress to competitive anxiety or from competitive anxiety to mental fatigue, and whether sleep quality, recovery behavior, coping flexibility, or contextual support explain how resilience reduces fatigue vulnerability. However, these models should be tested carefully because the present review does not claim that the proposed pathway has already been causally validated. Cross-sectional designs can identify associations, but they cannot establish temporal ordering, reciprocal effects, or causal direction.

Future empirical work should therefore prioritize longitudinal, multi-wave, daily-diary, or ecological momentary assessment designs that can capture how stress, anxiety, fatigue, resilience, and recovery fluctuate across real academic–athletic contexts. Such designs would allow researchers to test whether competitive anxiety contributes to later fatigue, whether mental fatigue subsequently increases anxiety vulnerability, and whether recovery opportunities interrupt this cycle. Combining self-report measures with training-load data, sleep indicators, cognitive tasks, brief daily fatigue ratings, or coach-reported functioning may also provide a more ecologically valid understanding of mental fatigue in collegiate athletes. These approaches would help transform the proposed conceptual framework into a testable empirical model while avoiding premature causal claims.

Overall, the current literature provides a strong basis for recognizing mental fatigue as an important issue in collegiate athletes, but further conceptual and methodological refinement is needed. Mental fatigue should be measured with greater precision, distinguished from related constructs, and studied within integrative frameworks that include stress appraisal, competitive anxiety, resilience-related regulation, and contextual support. Future research should prioritize multi-method measurement, longitudinal designs, culturally diverse samples, and intervention-oriented studies. These directions would strengthen both theoretical understanding and practical support for collegiate athletes managing sustained cognitive–emotional demands.

## 10. Practical Implications for Behavioral Intervention and Athlete Support

The framework developed in this review has practical implications for behavioral intervention, athlete support, coaching practice, and university sport systems. Mental fatigue should be recognized not only as a performance-related issue, but also as a cognitive–emotional regulation problem that may emerge from repeated stress appraisal, competitive anxiety, and insufficient recovery. If mental fatigue is misunderstood as laziness, weak motivation, or poor discipline, athletes may be blamed for symptoms that actually reflect accumulated psychological load and self-regulatory depletion. A behavioral science perspective therefore encourages coaches, sport psychologists, and university support staff to identify early signs of cognitive–emotional strain before these signs develop into more persistent fatigue-related impairment.

One important implication is the need for early monitoring of mental fatigue. Because mental fatigue may appear before obvious physical decline, support personnel should pay attention to indicators such as reduced concentration, slower tactical adjustment, irritability, increased perceived effort, emotional reactivity, lower motivation, and difficulty recovering from mistakes. These signs may be especially important in collegiate athletes because academic workload and competition pressure often accumulate across the same period. Monitoring should therefore include not only sport-related performance indicators, but also academic stress, sleep quality, recovery time, travel burden, and perceived coping capacity. Previous work on student–athlete mental health and well-being has emphasized that academic–athletic demands, service access, and help-seeking barriers are important factors in athlete support systems (Moreland et al., 2018; Cosh et al., 2024; Kegelaers et al., 2024).

A second implication concerns stress management. If perceived stress functions as an upstream appraisal-based antecedent of mental fatigue, then interventions should target how athletes interpret and manage demands. Athletes may benefit from psychoeducation that helps them distinguish between manageable challenge, harmful overload, and early signs of cognitive depletion. Cognitive restructuring, stress appraisal training, time-management support, and problem-solving strategies may help athletes reduce the extent to which academic and athletic demands are interpreted as uncontrollable. This is consistent with the Transactional Model of Stress and Coping, which emphasizes that stress responses depend on both primary appraisal of demands and secondary appraisal of coping resources (Lazarus & Folkman, 1984). In practical terms, interventions should not only reduce demands where possible, but also strengthen athletes’ perceived ability to manage them.

A third implication relates to competitive anxiety regulation. Because competitive anxiety may operate as a proximal emotional mechanism linking perceived stress to mental fatigue, interventions should help athletes manage worry, arousal, self-monitoring, and fear of failure in evaluative situations. Techniques such as relaxation training, controlled breathing, attentional refocusing, cognitive reappraisal, imagery, pre-performance routines, and self-talk may reduce the regulatory cost of anxiety during performance. Psychological skill training has been widely discussed as a way to improve performance and well-being in high-intensity sport environments, especially when it targets confidence, attention, arousal regulation, and coping under pressure (Birrer & Morgan, 2010; Golby & Wood, 2016). These strategies may help athletes prevent temporary anxiety from escalating into sustained cognitive–emotional depletion.

A fourth implication is the need to strengthen resilience as a developable regulatory resource. Resilience should not be treated as a fixed quality that athletes either possess or lack. Instead, it can be developed through repeated coping experiences, supportive coaching, reflective practice, psychological skill training, and recovery-focused routines. Athletes with stronger resilience may be better able to reappraise stressors, maintain confidence after mistakes, regulate emotional responses, and recover from performance setbacks. This interpretation is consistent with sport resilience research that views resilience as a dynamic process shaped by personal resources, adversity exposure, coping strategies, and supportive environments (Fletcher & Sarkar, 2012, 2013; Sarkar & Fletcher, 2014). Therefore, resilience-building interventions should combine individual psychological training with broader environmental support.

Recovery behavior is another important area for practical application. Mental fatigue is not only caused by the presence of demands, but also by insufficient restoration after those demands. According to Conservation of Resources theory, stress becomes harmful when valued resources such as energy, attention, confidence, emotional stability, and support are threatened or depleted without adequate replenishment (Hobfoll, 1989). For collegiate athletes, recovery should therefore include psychological as well as physical components. Brief recovery routines, sleep hygiene, structured breaks from academic and sport demands, post-competition emotional decompression, mindfulness-based strategies, and deliberate detachment from performance evaluation may all help restore self-regulatory resources. Recovery planning should be integrated into training schedules rather than treated as an optional addition.

At the coaching level, the framework suggests that communication style and performance climate matter. Coaches who respond to mistakes with excessive criticism may intensify athletes’ fear of failure, self-monitoring, and competitive anxiety. By contrast, coaches who provide clear feedback, normalize mistakes as part of learning, and encourage adaptive reflection may reduce the psychological cost of performance evaluation. Supportive coach-athlete relationships may also make it easier for athletes to disclose stress or fatigue before problems become severe. This is particularly important because many athletes may hide psychological strain due to stigma, fear of losing selection opportunities, or concerns about appearing mentally weak. Reviews of athlete help-seeking have identified stigma, confidentiality concerns, and limited awareness of services as important barriers to psychological support (Moreland et al., 2018; Cosh et al., 2024).

At the institutional level, universities should recognize mental fatigue as both a performance issue and a student well-being issue. Collegiate athletes are not only performers; they are also students managing academic obligations, identity development, and future career concerns. University sport systems can reduce fatigue vulnerability by providing academic flexibility during competition periods, coordinated communication between academic and athletic departments, accessible sport psychology services, mental health literacy programs, and reasonable travel and recovery schedules. Such structural supports are especially important because resilience should not be framed only as individual responsibility. If institutional environments repeatedly create overload without sufficient support, athletes may experience resource depletion regardless of personal coping capacity.

Collegiate tennis provides a useful example of how these implications may be applied. Tennis players often need to regulate emotions independently between points, recover from errors without continuous team support, and sustain tactical decision-making across changing score conditions. Interventions such as between-point routines, attentional reset strategies, post-error recovery scripts, and confidence-focused self-talk may therefore be especially useful in this context. However, the broader implications extend beyond tennis. Any collegiate sport that requires sustained attention, emotional regulation, repeated evaluation, and rapid decision-making may benefit from interventions targeting stress appraisal, competitive anxiety, resilience, and recovery behavior.

Overall, the practical value of the proposed framework lies in its emphasis on multi-level prevention and regulation rather than only post-problem intervention. At the individual level, collegiate athletes may benefit from regular monitoring of concentration difficulties, perceived effort, emotional reactivity, sleep quality, recovery opportunity, and anxiety-related self-monitoring. At the coaching level, supportive communication, adaptive feedback after mistakes, structured recovery routines, and normalization of psychological help-seeking may reduce the likelihood that stress and competitive anxiety develop into sustained mental fatigue. At the institutional level, universities can support athlete well-being by improving coordination between academic and athletic departments, offering flexible academic arrangements during intensive competition periods, providing accessible sport psychology services, and integrating mental health literacy into athlete development programs.

These recommendations should be interpreted as theoretically informed implications derived from the conceptual framework rather than as a fully validated intervention protocol. Because the present review is a structured narrative and conceptual synthesis, future intervention studies are needed to test whether programs targeting stress appraisal, competitive anxiety regulation, resilience development, recovery planning, and contextual support can reduce mental fatigue in collegiate athletes. Nevertheless, the framework suggests that athlete support programs may be more effective when they address perceived stress, competitive anxiety, psychological resilience, recovery behavior, and institutional environment together rather than treating mental fatigue as an isolated individual weakness.

More specifically, coaches and support staff can monitor early indicators of mental fatigue, including reduced concentration, slower tactical adjustment, increased perceived effort, irritability, and difficulty recovering from mistakes. Sport psychologists can address stress appraisal, anxiety-related self-monitoring, attentional refocusing, and recovery routines through psychoeducation, cognitive reappraisal, breathing strategies, pre-performance routines, and post-performance reflection. University sport systems can support athletes by coordinating academic and training schedules, protecting sleep and recovery opportunities, providing accessible psychological services, and reducing help-seeking barriers. These actions should be interpreted as theoretically informed recommendations that require future intervention testing rather than as a validated treatment protocol.

## 11. Conclusions

Mental fatigue warrants greater attention in collegiate athletes because it occupies the intersection of cognitive strain, emotional pressure, self-regulation, recovery, and performance sustainability. Rather than being understood as simple tiredness or only as a secondary consequence of physical exertion, mental fatigue should be conceptualized as a cognitive–emotional and psychobiological outcome shaped by repeated exposure to academic, competitive, and psychosocial demands. The literature reviewed in this article suggests that fatigue-related vulnerability may emerge when sustained cognitive load, stress appraisal, competitive anxiety, insufficient recovery, and resource depletion occur together (Marcora et al., 2009; Van Cutsem et al., 2017; Pageaux & Lepers, 2018). This perspective is important because collegiate athletes may remain physically capable while still experiencing impaired concentration, increased perceived effort, emotional reactivity, reduced decision quality, and weakened behavioral consistency.

The central contribution of this review is that it organizes fragmented literature on perceived stress, competitive anxiety, psychological resilience, recovery, contextual support, and mental fatigue into a testable conceptual framework for collegiate athletes. The framework does not demonstrate that stress causes anxiety or that anxiety causes mental fatigue. Rather, it identifies plausible relationships that may be examined in future longitudinal, daily-diary, ecological momentary assessment, and intervention studies. By maintaining this distinction between evidence, adjacent support, and theoretical inference, this review offers a cautious foundation for future research and applied support in collegiate sport.

At the same time, the proposed framework should be interpreted cautiously. This review does not claim that the stress–anxiety–fatigue pathway has already been causally validated. Instead, it organizes existing direct evidence, adjacent evidence, and theoretical inference into a conceptual heuristic framework for future empirical testing. The framework also recognizes that the pathway may be reciprocal rather than strictly one-directional. Competitive anxiety may contribute to mental fatigue by increasing attentional disruption, emotional regulation demands, and perceived effort, but mental fatigue may also increase later anxiety vulnerability by weakening attentional control, emotional stability, and confidence. This reciprocal interpretation is especially relevant in collegiate sport, where academic workload, training demands, competition pressure, sleep disruption, and insufficient recovery may accumulate across repeated performance cycles.

This review also clarifies that resilience should not be romanticized as invulnerability or treated only as an individual personality trait. Resilient athletes may still experience stress, anxiety, frustration, and temporary cognitive strain. The protective value of resilience lies not in eliminating pressure, but in helping athletes regulate its consequences and recover before temporary strain develops into sustained fatigue-related impairment. Viewing resilience as a dynamic regulatory resource highlights the importance of coping strategies, coaching relationships, institutional support, recovery opportunities, and help-seeking environments (Fletcher & Sarkar, 2012, 2013; Sarkar & Fletcher, 2014). This broader understanding is particularly relevant in collegiate sport, where athletes’ psychological functioning is shaped by both personal resources and university systems.

This review further highlights several limitations in the current literature. Mental fatigue remains inconsistently defined and measured, with studies relying on diverse indicators such as self-report symptoms, perceived effort, cognitive tasks, physiological markers, and sport-performance outcomes. In addition, many studies examine perceived stress, competitive anxiety, resilience, and fatigue separately rather than as linked psychological processes. Much of the available evidence is also cross-sectional or short-term, limiting understanding of temporal ordering, reciprocal effects, and causal direction. More culturally diverse and non-Western collegiate athlete research is needed because academic expectations, family pressure, institutional structures, coaching climates, and help-seeking norms may shape stress appraisal, anxiety regulation, resilience development, and fatigue recovery.

Future research should therefore adopt more precise, integrative, and context-sensitive approaches. Multi-method measurement strategies are needed to distinguish mental fatigue from adjacent constructs such as burnout, general tiredness, emotional exhaustion, and overtraining. Longitudinal, multi-wave, daily-diary, ecological momentary assessment, and intervention-based designs are also needed to test whether perceived stress predicts later competitive anxiety, whether competitive anxiety contributes to accumulated mental fatigue, whether mental fatigue subsequently increases anxiety vulnerability, and whether resilience, sleep, recovery behavior, or contextual support moderates these pathways. Such approaches would help transform the proposed conceptual framework into a testable empirical model while avoiding premature causal claims.

From an applied perspective, mental fatigue should be treated as both a performance issue and a student well-being issue. Coaches, sport psychologists, and university support systems should monitor early signs of cognitive–emotional strain, reduce avoidable overload, strengthen athletes’ coping and resilience resources, and create environments that support recovery and help-seeking. Interventions should not address perceived stress, competitive anxiety, resilience, and mental fatigue as separate problems. Instead, athlete support should target the broader pathway through which demands are appraised, anxiety is regulated, resources are depleted or restored, and fatigue-related vulnerability develops. Psychological skill training, stress appraisal education, attentional control strategies, emotional recovery routines, sleep and recovery planning, and accessible mental health services may all contribute to more sustainable athlete functioning.

Collegiate tennis provides a useful applied example because it requires sustained concentration, individualized accountability, rapid tactical decision-making, and repeated emotional recovery. However, the framework proposed in this review is not limited to tennis. Tennis and Chinese/non-Western collegiate sport contexts are used as illustrative applications rather than exclusive empirical targets. By reframing mental fatigue as a behavioral science issue involving stress appraisal, competitive anxiety, cognitive–emotional regulation, resilience, recovery, and contextual support, this review offers a foundation for future theory development, empirical testing, and intervention design. A more integrated understanding of these processes may help universities and sport systems support not only athlete performance, but also long-term psychological well-being and sustainable development.

## Figures and Tables

**Table 1:** Search strategy, eligibility logic, and synthesis procedure.

Review Component	Description
Review design	Structured narrative review and conceptual synthesis
Main purpose	To integrate literature on mental fatigue, perceived stress, competitive anxiety, and psychological resilience in collegiate athletes
Databases searched	Scopus, Web of Science, PubMed, and Google Scholar
Search period	Literature published up to February 2026
Core search terms	“mental fatigue”, “cognitive fatigue”, “collegiate athletes”, “student-athletes”, “perceived stress”, “stress appraisal”, “competitive anxiety”, “sport anxiety”, “psychological resilience”, “coping”, “self-regulation”, “emotion regulation”, “athlete mental health”, “sport performance”
Additional contextual terms	“tennis”, “racket sport”, “individual sport”, “Chinese collegiate athletes”, “non-Western athletes”, “academic-athletic role conflict”, “sleep”, “recovery”, “burnout”, “help-seeking”, “institutional support”
Inclusion logic	Sources were included when they clarified mental fatigue, stress appraisal, competitive anxiety, resilience, coping, recovery, measurement issues, cultural context, or collegiate athlete well-being
Exclusion logic	Sources were not prioritized when they focused on unrelated clinical, occupational, or non-athlete populations without clear relevance to sport, performance, stress, anxiety, resilience, or fatigue
Types of evidence prioritized	Peer-reviewed empirical studies, theoretical papers, systematic reviews, meta-analyses, and conceptual reviews
Use of tennis context	Tennis was used as an illustrative applied context because it involves sustained attention, individual accountability, rapid tactical decision-making, and emotional recovery
Use of Chinese/non-Western context	Chinese and non-Western collegiate sport settings were used to illustrate how cultural expectations, academic pressure, institutional support, and help-seeking norms may shape fatigue processes
Synthesis procedure	Sources were grouped thematically according to conceptual definitions, stress appraisal, competitive anxiety, resilience, recovery, measurement limitations, cultural context, and practical implications
Limitation of approach	This review did not use PRISMA screening, formal risk-of-bias assessment, or meta-analysis; therefore, it should be interpreted as a transparent narrative synthesis rather than an exhaustive evidence map

**Table 2:** Evidence status of the proposed pathways in the behavioral mechanism framework.

Proposed Pathway or Construct Relationship	Current Evidence Status	Interpretation for the Present Review	Future Research Implication
Academic–athletic demands → perceived stress	Supported by collegiate athlete and student–athlete literature	Academic workload, training demands, competition schedules, travel, role conflict, and institutional expectations can contribute to perceived stress in collegiate athletes.	Future studies should examine how academic and athletic demands accumulate across semesters, training cycles, and competition periods.
Perceived stress → competitive anxiety	Supported by broader stress and sport anxiety literature, with partial evidence in athlete populations	Perceived stress may increase vulnerability to worry, self-doubt, threat appraisal, and anxiety in evaluative performance situations.	Longitudinal studies should test whether perceived stress predicts later competitive anxiety in collegiate athletes.
Competitive anxiety → mental fatigue	Supported mainly by adjacent evidence and theoretical inference	Competitive anxiety may increase attentional disruption, self-monitoring, emotional regulation demands, and perceived effort, thereby contributing to fatigue-related impairment.	Experimental and longitudinal studies should test whether competitive anxiety predicts mental fatigue through attentional and self-regulatory mechanisms.
Perceived stress → mental fatigue	Supported by stress, depletion, and fatigue literature, but less directly tested in collegiate athlete samples	Repeated stress appraisal may consume cognitive and emotional resources and increase vulnerability to mental fatigue.	Future research should test direct and indirect pathways from perceived stress to mental fatigue using multi-wave or daily-diary designs.
Psychological resilience moderating perceived stress → competitive anxiety	Supported by resilience and coping theory, with partial evidence in sport psychology	Resilience may influence how athletes appraise stressors and evaluate their coping resources, reducing the likelihood that stress develops into anxiety.	Studies should examine whether resilience moderates stress–anxiety associations in collegiate athlete populations.
Psychological resilience moderating competitive anxiety → mental fatigue	Mainly theoretical and supported by adjacent resilience literature	Resilience may support emotional recovery, attentional refocusing, coping flexibility, and confidence maintenance, thereby weakening anxiety-related fatigue vulnerability.	Future studies should test resilience as a moderator between competitive anxiety and mental fatigue.
Sleep and recovery → mental fatigue	Supported by recovery, sleep, and athlete well-being literature	Insufficient recovery may intensify cognitive–emotional depletion and reduce athletes’ ability to restore attentional and emotional resources.	Future models should include sleep quality, recovery behavior, and recovery opportunity as mediators or moderators.
Mental fatigue → competitive anxiety	Theoretical reciprocal pathway supported indirectly by anxiety and fatigue literature	Mental fatigue may also increase anxiety by weakening attentional control, emotional stability, and confidence during performance.	Future longitudinal studies should test reciprocal relationships rather than assuming a one-directional pathway.
Contextual support → stress, resilience, and recovery	Supported by athlete mental health, help-seeking, and sport environment literature	Coaching climate, institutional support, academic flexibility, psychological services, and cultural norms may shape stress appraisal, resilience development, and recovery opportunities.	Future research should examine contextual and cultural moderators, especially in non-Western collegiate sport settings.
Tennis and Chinese/non-Western collegiate contexts as illustrative applications	Contextually relevant but not exclusive empirical targets	Tennis illustrates individual accountability, rapid decision-making, and emotional recovery demands, while Chinese/non-Western contexts illustrate cultural and institutional influences on stress and support.	Future studies should test the framework across different sports, cultures, and university systems.

Note. The arrow (→) indicates the proposed direction of the pathway or construct relationship.

## Data Availability

No new data were generated or analyzed in this study. This manuscript is a structured narrative review based on published literature and conceptual synthesis.
